# Histological evaluation of AMPK signalling in primary breast cancer

**DOI:** 10.1186/1471-2407-9-307

**Published:** 2009-09-01

**Authors:** Sirwan M Hadad, Lee Baker, Philip R Quinlan, Katherine E Robertson, Susan E Bray, George Thomson, David Kellock, Lee B Jordan, Colin A Purdie, David G Hardie, Stewart Fleming, Alastair M Thompson

**Affiliations:** 1 Department of Surgery and Molecular Oncology, Ninewells Hospital and Medical School, Dundee, UK; 2 Department of Pathology and Neuroscience, Ninewells Hospital and Medical School, Dundee, UK; 3 Division of Molecular Physiology, College of Life Sciences, University of Dundee, Dundee, UK

## Abstract

**Background:**

AMP-activated protein kinase (AMPK) acts as a cellular fuel gauge that responds to energy stress by suppressing cell growth and biosynthetic processes, thus ensuring that energy-consuming processes proceed only if there are sufficient metabolic resources. Malfunction of the AMPK pathway may allow cancer cells to undergo uncontrolled proliferation irrespective of their molecular energy levels. The aim of this study was to examine the state of AMPK phosphorylation histologically in primary breast cancer in relation to clinical and pathological parameters.

**Methods:**

Immunohistochemistry was performed using antibodies to phospho-AMPK (pAMPK), phospho-Acetyl Co-A Carboxylase (pACC) an established target for AMPK, HER2, ERα, and Ki67 on Tissue Micro-Array (TMA) slides of two cohorts of 117 and 237 primary breast cancers. The quick score method was used for scoring and patterns of protein expression were compared with clinical and pathological data, including a minimum 5 years follow up.

**Results:**

Reduced signal, compared with the strong expression in normal breast epithelium, using a pAMPK antibody was demonstrated in 101/113 (89.4%) and 217/236 (91.9%) of two cohorts of patients. pACC was significantly associated with pAMPK expression (p = 0.007 & p = 0.014 respectively). For both cohorts, reduced pAMPK signal was significantly associated with higher histological grade (p = 0.010 & p = 0.021 respectively) and axillary node metastasis (p = 0.061 & p = 0.039 respectively). No significant association was found between pAMPK and any of HER2, ERα, or Ki67 expression, disease-free survival or overall survival.

**Conclusion:**

This study extends *in vitro *evidence through immunohistochemistry to confirm that AMPK is dysfunctional in primary breast cancer. Reduced signalling via the AMPK pathway, and the inverse relationship with histological grade and axillary node metastasis, suggests that AMPK re-activation could have therapeutic potential in breast cancer.

## Background

AMP-activated protein kinase (AMPK) is an intracellular energy-sensing kinase that is inactive unless it has been phosphorylated by upstream kinases at a specific threonine residue (Thr-172) within the kinase domain. Phosphorylation at Thr-172 and consequent activation occurs in response to metabolic stresses that deplete cellular energy levels and thus increase the AMP/ATP ratio [1,2]. Activation of AMPK can be assessed using a phosphospecific antibody (anti-pAMPK) that recognizes either catalytic subunit isoform (α1 or α2), but only when phosphorylated at Thr-172. AMPK is proposed as a "fuel gauge" that monitors changes in the energy status of cells [2,3]. It is activated by metabolic stresses that inhibit ATP production such as glucose deprivation [4], ischaemia [5], or hypoxia [6], or by stresses that accelerate ATP consumption such as contraction in skeletal muscle [7]. Once activated, AMPK down-regulates energy-consuming processes, including cell proliferation, thus ensuring that these processes only proceed if there are sufficient metabolic resources available [8]. AMPK switches on catabolic pathways that generate ATP, while switching off biosynthetic pathways and other processes that consume ATP, and hence has a key role in maintaining energy balance both at the single cell and the whole body levels [2,9].

One direct downstream target of AMPK is acetyl-Coenzyme A carboxylase (ACC). In its active, de-phosphorylated form, ACC catalyzes the conversion of acetyl-CoA to malonyl-CoA in the de novo lipid synthesis pathway [3,10,11]. When it is inactivated by phosphorylation, a decrease in malonyl-CoA occurs, thereby increasing the mitochondrial import and oxidation of long chain fatty acids (LCFAs), resulting in the generation of ATP [12]. ACC exists as two isoforms, ACC1/a and ACC2/b, with ACC1 being thought to produce the pool of malonyl-CoA involved in fatty acid synthesis, while ACC2 is thought to produce the mitochondrial pool that regulates fatty acid oxidation [13,14]. AMPK phosphorylates ACC1 at multiple residues, although phosphorylation at a single serine (Ser-79 in rat and Ser-80 in human ACC1) accounts for the resulting inactivation [15,16]. AMPK also inactivates ACC2 [17] via phosphorylation at the site equivalent to Ser-79 on ACC1 (Ser-221 in human ACC2). A phosphospecific antibody (anti-pACC) recognizes both ACC1 and ACC2 phosphorylated at these sites, and is a widely used marker for AMPK activation. In isolated hepatocytes from AMPK-α1^-/- ^and -α2^-/- ^double knockout mice, the signal obtained using this antibody is completely lost, showing that AMPK is responsible for phosphorylation at these sites [18].

There is a large body of evidence to support the link between metabolic disorders, such as obesity and type 2 diabetes, and dysfunctional lipid and energy metabolism causing increases in circulatory and intracellular fatty acids [10,12,19]. High levels of fatty acids are toxic to cells and may cause deleterious metabolic abnormalities [12,19]. These unwanted effects could be prevented by activation of AMPK and consequent inactivation of ACC2 in peripheral tissues, leading to an increase in fatty acid oxidation [12,19]. In addition many cancer cells, including many breast tumours, exhibit a markedly increased rate of fatty acid synthesis [20]. Some breast tumours express high levels of fatty acid synthase (FAS), a key enzyme for fatty acid biosynthesis [21]. FAS is up-regulated in about 50% of breast cancers, is an indicator of poor prognosis, and is associated with the HER2 oncogene [22-24]. The drug Orlistat (Xenical™), which was developed as an inhibitor of gastric and pancreatic lipases and has been approved for weight loss by the FDA, blocks breast cancer cell cycle progression, promotes apoptotic cell death, and transcriptional repression of the HER2 oncogene [25]. In addition to these potentially beneficial effects of AMPK activation on lipid metabolism, AMPK would also be expected to directly inhibit cell growth and proliferation. The major kinase that phosphorylates Thr-172 on AMPK has been identified to be the tumour suppressor kinase, LKB1 [26,27]. Although LKB1 is also the upstream kinase for a family of twelve additional AMPK-related kinases [28,29], many of the tumour suppressor effects of LKB1 are likely to be mediated by AMPK. For example, AMPK activation causes a G1-S phase cell cycle arrest [30,31], while it also inhibits cell growth by switching off the target-of-rapamycin (TOR) pathway [32,33]. Consistent with the idea that AMPK activation might inhibit growth of tumour cells were findings that the oral anti-diabetic drug, metformin, inhibits proliferation of epithelial cells derived from breast, prostate and ovarian cancers, an effect that requires both LKB1 and AMPK [34]. These findings may be clinically relevant, because epidemiological studies show an association between a lower incidence of cancer, including breast cancer, and metformin usage in diabetic patients [35,36].

Despite growing evidence in this arena, little is known about the state of phosphorylation of AMPK, and its downstream target ACC, in human breast cancers. Since malignant cells are likely to have an increased energy demand and may also experience hypoxia and other metabolic stresses, one might speculate that AMPK signalling would be activated. The objective of the present study was to evaluate the status of phosphorylation of AMPK and ACC signalling in human primary breast cancer using immunohistochemistry, and to correlate this with the clinical and pathological characteristics of the tumours.

## Methods

### Patients

A total of 354 primary human breast cancer specimens were used in this study from two cohorts of patients. The first cohort comprised 117 of the pre- and post-menopausal women (aged 34 - 76; median 51 years) with primary breast cancer who participated in the Adjuvant Breast Cancer (ABC) clinical trial from 1992 to 2000 at a single recruiting centre (NHS Tayside) [37].

The second cohort of patients comprised 237 unselected pre- and post-menopausal women (aged 28 - 89; median 62 years) with primary, previously untreated breast cancer who were seen and treated at Tayside University Hospitals, Scotland, from 1997 to 2002. Informed consent was obtained from each patient prior to tissue acquisition. Tumour samples from these patients were constructed on tissue microarray (TMA) slides as described below. Ethics approval was given by the Tayside Research and Ethics committee (Ref. 07/S1402/90).

### TMA construction

A TMA containing up to six 0.6 mm diameter cores of invasive tumours and normal breast tissue from the same patients was constructed using a manual tissue arrayer (Beecher Instruments Inc., Sun Prairie, WI, USA). Briefly, haematoxylin and eosin (H&E) stained sections of normal and tumour tissues were reviewed by a single pathologist. Areas suitable for inclusion in the TMA were marked on the slide. Marked slides were matched to their corresponding wax blocks (the donor blocks). The areas of interest were marked on the wax blocks using a marker pen and 0.6 mm diameter cores of tumour or normal breast tissue were removed and inserted into a pre-cored hole in a recipient paraffin block. Cores were inserted in a grid arrangement. Normal breast tissue cores were constructed alongside the cancerous cores for every patient. Four micron TMA sections were cut, mounted onto poly-L-lysine coated glass slides (VWR International Ltd., Lutterworth, UK), and dried for 1 hour at 60°C before being de-paraffinised in Histoclear (National Diagnostics, Hessle, UK) and xylene, then run through a graded series of alcohol and rehydrated under running water.

### Immunohistochemistry (IHC)

pAMPK (Thr-172) monoclonal antibody and pACC (Ser-79) were purchased from Cell Signaling Technology. MIB1 (Anti-Ki67) and anti-HER2 monoclonal antibodies were purchased from Novocastra. Optimal staining conditions such as epitope unmasking, antibody titre, and incubation and visualisation method were validated on conventional whole tissue sections and TMA fragments. Positive controls included normal spleen and breast epithelium for pAMPK and pACC, and breast cancer samples of established ER, HER2 and Ki67. Citric acid buffer, pH 6.0 (10 mM) was used as standard microwave-based antigen retrieval method for all proteins except for pACC. Sections were immersed in 10 mM Citric acid buffer, pH 6.0 and subjected to microwave in a pressure vessel for 15 min before then processed on a DAKO autostainer using Vectastatin^® ^ABC kits (Vector Labs, Peterborough, UK) according to the manufacturer's protocol. Ethylene diaminetetraacetic acid disodium salt dihydrate (EDTA) (Sigma product E-5134) for 45 minutes at 94°C was used for pACC antigen retrieval. Sections were blocked by either normal goat, horse or rabbit serum containing 10% (v/v) from stock avidin solution (Vector Labs) for 20 min followed by incubation with primary antibody including 10% (v/v) from stock biotin solution (Vector Labs) for 1 hour to reduce non-specific background staining. Following incubation with the specific primary antibodies, sections were incubated with either biotinylated anti-rabbit, anti-sheep, or anti-mouse antibody for 30 min followed by Vectastain^® ^Elite ABC reagent for another 30 min. Liquid diaminobenzidine (DAB; Dako) was used as a chromogenic agent for 5 min and sections were counter-stained with Mayer's haematoxylin. In between each immunostaining step, slides were washed briefly in Tris buffered saline (TBS) buffer, pH 7.6.

### Scoring

**Figure 1:**
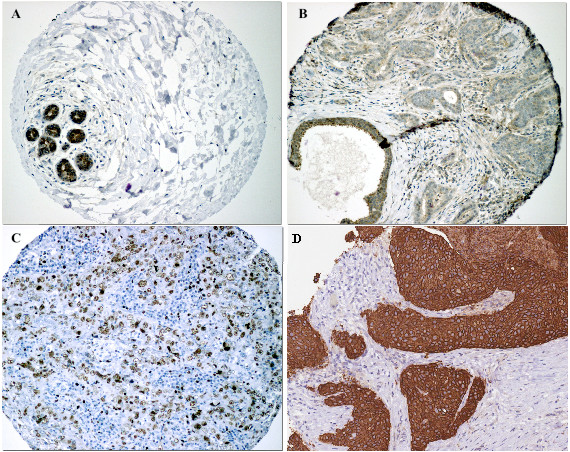
**Strong staining of AMPK in normal breast lobular unit (A) compared to its light staining in tumour cells (B), examples of a positive Ki67 (C) and HER2 (D).**

Primary TMA scoring was performed by a single author (SMH), verified by an experienced consultant pathologist (SF) and where needed by a specialist breast pathologist (LJ or CAP) using a Nikon Eclipse E600 light microscope with DXM 1200 digital camera and Eclipse-Net software, and Virtual Microscopy in the form of Aperio Technologies, ScanScope XT and Spectrum Plus using TMA software version 9.1. Cores were scored at a magnification of ×40 (Figure 1). Any discrepancies were resolved by subsequent consultation. HER2 immunostaining of cores containing tumour were evaluated as 0, 1+, 2+ and 3+ according to the US FDA-approved scoring guidelines for the Hercep Test [52]: non-specific membrane staining = 0, membrane or cytoplasmic staining in <10% of cells = 1+, specific membrane staining in >10% of cells = 2+, and strong specific staining in majority of cells = 3+. HER2 amplification was confirmed for 2+ and 3+ tumours using FISH (Fluorescence In Situ Hybridization). For Ki67 proliferation index (the fraction of proliferating cells), ERα, pAMPK and pACC antibody staining of cores containing tumour were assessed using a scoring system based on the Quick Score Method [35]. Immuno-reactivity scored semi-quantitatively for both the intensity and the proportion of cells staining: intensity was given scores 0-3 (no staining = 0; light staining = 1; moderate staining = 2; strong staining = 3) and proportion was given scores 1-6 (0-4% = 1; 5-20% = 2; 21-40% = 3; 41-60% = 4; 61-80% = 5; 81-100% = 6). The two scores were then multiplied to obtain the final result of 0 - 18. Cores from normal spleen and normal breast tissue were used as control on each slide.

### Statistics

All analyses were performed using Microsoft Excel 2002 SP3 (Microsoft Corporation) and Minitab Release 14.13 (Minitab Inc.). Appropriate choice of *χ*^2 ^for trend and Fisher's Exact Test (FET) were used for significance of expression, and non-parametric survival plots used the Kaplan-Meier method. When grouping of scores was required for a statistical method, such as Fisher's Exact Test, pAMPK scores were grouped as scores 3 versus scores 0 - 2, chosen because normal breast epithelial cell staining had similar strong staining as the tumour cells scored 3. The pAMPK results were based on comparing strongly positive AMPK signalling in tumour cells that scored 3 to scores of 0 - 2. For Ki67, scores 0 - 2 were grouped against 3 - 6. For HER2, scores 0 - 1 versus 2 - 3 with FISH verification of the amplification of the latter group; while for ERα, scores 0 - 3 (negative) versus 4 - 18 (positive) was chosen [38]. However, where *χ*^2 ^for trend was used, the scores were not dichotomised. The null hypothesis was rejected at an α level of 10% (p ≤ 0.10), and observations considered to be marginal (i.e. of scientific interest and worthy of further investigation) for an α level between 5% and 10% (p ≤ 0.10) and significant at 5% (p ≤ 0.05).

## Results

For the first cohort of the 117 patients, 104 (88.9%) were infiltrating ductal carcinoma, 3 (2.6%) infiltrating lobular and 10 (8.5%) mixed or other types. All cases were successfully scored for Ki67 and HER2; 3/117 (2.6%) for pACC, and 4/117 (3.4%) for pAMPK were not evaluable on the TMAs.

In the second cohort of 237 patients, 197 (83.1%) were infiltrating ductal carcinoma, 16 (6.7%) infiltrating lobular carcinoma, and 34 (14.3%) were mixed and other types. One case (1/237 (0.4%)) was excluded from scoring for pAMPK and 19/237 (8%) for pACC were not evaluable (Table 1).

**Table 1:** Clinico-pathological characteristics of the patients.

Parameter	Cohort 1 [n (%)]	Cohort 2 [n (%)]	P
***N***	117	237	

***Menopausal status at diagnosis***			

Pre- and peri-menopausal	62 (53)	56 (24)	< 0.001*
	
Post-menopausal	55 (47)	181 (76)	

***Age (years)***			

Median (range)	51 (34-76)	62 (28-89)	< 0.001^~^

***Tumour size (cm)***			

≤ 2	61 (57)	88 (37)	< 0.001*
	
> 2	46 (43)	148 (63)	

***Number of positive lymph nodes***			

0	48 (41)	127 (54)	0.032*^†^ 0.540*^‡^
	
1-3	48 (41)	74 (31)	
	
> 3	21 (18)	36 (15)	

***Histological grade***			

1	12 (10)	34 (14)	0.399*^†^ 0.425*^‡^
	
2	43 (38)	91 (39)	
	
3	59 (52)	110 (47)	

***Adjuvant therapy***			

Endocrine therapy	18 (16)	59 (27)	N/A
	
Chemotherapy and/or Radiotherapy	0 (0)	54 (24)	
	
Endocrine + Chemotherapy	12 (10)	10 (4)	
	
Endocrine + Radiotherapy	45 (38)	70 (31)	
	
Endocrine + Chemo + Radiotherapy	42 (36)	31 (14)	

***Follow up (months)***			
	
Median (range)	73 (9-122)	60 (1-120)	< 0.001^~^

Notes: *2-sided Fisher's Exact Test; ^~ ^Mann-Whitney U-test; ^†^first row *versus *summation of all other rows; ^‡^last row *versus *summation of all other rows.

Cytoplasmic staining was seen for pAMPK and, as expected, nuclear staining for Ki67 and ERα; combined cell membrane and cytoplasm staining for pACC; cell membrane strongly and cytoplasm lightly for HER2. Normal breast epithelium did not stain for Ki67, HER2 or pACC; however strong staining of normal breast epithelial cytoplasm was observed for pAMPK (Figure 1A).

Reduced AMPK phosphorylation signal (scores 0 - 2) was seen in the vast majority of the breast cancer specimens (101/113 [89.4%] and 217/236 [91.9%] of the first and second cohort respectively), as light staining of the cytoplasm (Figure 1B), compared to strong expression in normal breast epithelium (score 3). A significant positive association was seen between pAMPK and pACC signals (FET: p = 0.007 and 0.014 respectively). Reduced AMPK phosphorylation signal was associated with higher histological grade (*χ*^2 ^for trend = 6.644, df = 1, p = 0.010; and *χ*^2 ^for trend = 5.291, df = 1, p = 0.021). AMPK phosphorylation signal reduction was also associated with axillary node metastasis (FET: p = 0.061 and 0.039 respectively) (Table 2 and additional file 1)

**Table 2:** The association between AMPK and all clinico-pathological and immunohistochemical data

		Cohort 1			Cohort 2		
**Parameter**		**Positive**	**Negative**	**P**	**Positive**	**Negative**	**P**

**HER2**	Positive	0	14	NS	0	13	NS
	Negative	12	87		3	95	

**Ki67**	Positive	4	36	NS	7	95	NS
	Negative	8	65		11	111	

**ACC**	Positive	7	20	0.007	7	29	0.014
	Negative	5	81		12	186	

**ERα**	Positive	9	61	NS	12	157	NS
	Negative	3	38		7	59	

**Tumour size (cm)**	≤ 2	7	51	NS	9	79	NS
	> 2	2	43		10	138	

**Positive axillary nodes**	0	8	39	0.021	15	111	0.087
	1-3	2	44		2	71	
	> 3	0	12		2	35	

**Histological grade**	1	5	7	0.010	6	57	0.021
	2	2	39		7	81	
	3	4	53		5	99	

**KM overall survival**	Alive	11	73	NS	11	154	NS
	Dead	1	28		4	35	

**Figure 2:**
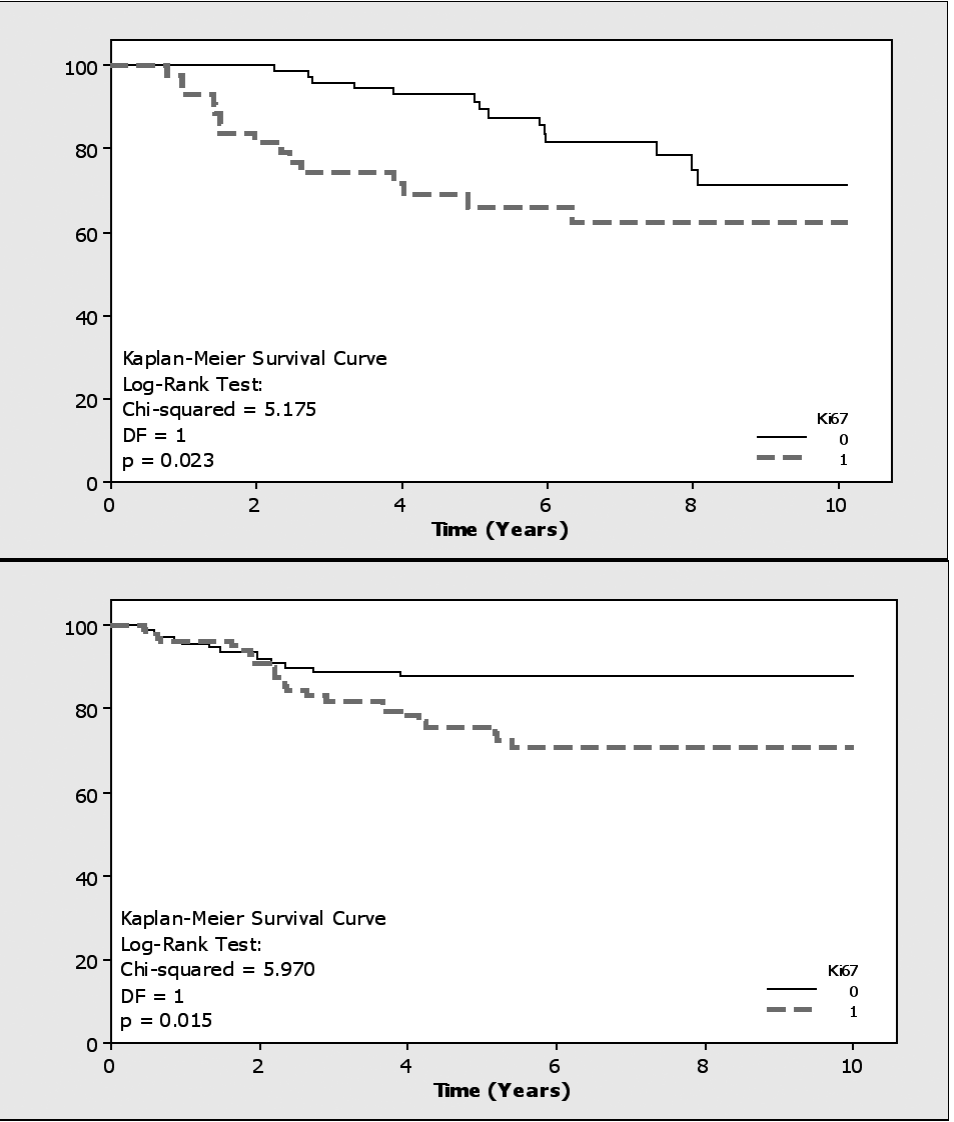
**Kaplan-Meier Survival analysis correlating Ki67 expression to overall survival in both cohorts (p = 0.023 and p = 0.015 respectively)**.

Ki67 expression (Figure 1C) was negatively associated with ER status (FET: p = 0.002 and p < 0.001 respectively) (see additional file 1). It was also associated with higher grade tumours (FET: p < 0.001 in both cohorts), increased tumour recurrence (FET: p = 0.041 and 0.005, Relative Risk (RR) = 1.89 and 1.59, Odds Ratio (OR) = 2.39 and 2.47, respectively) and lower overall survival (Kaplan-Meier Log Rank Test: p = 0.023 and 0.015, RR = 1.62 and 2.13, OR = 2.29 and 23.39, respectively) (Figure 2).

**Figure 3:**
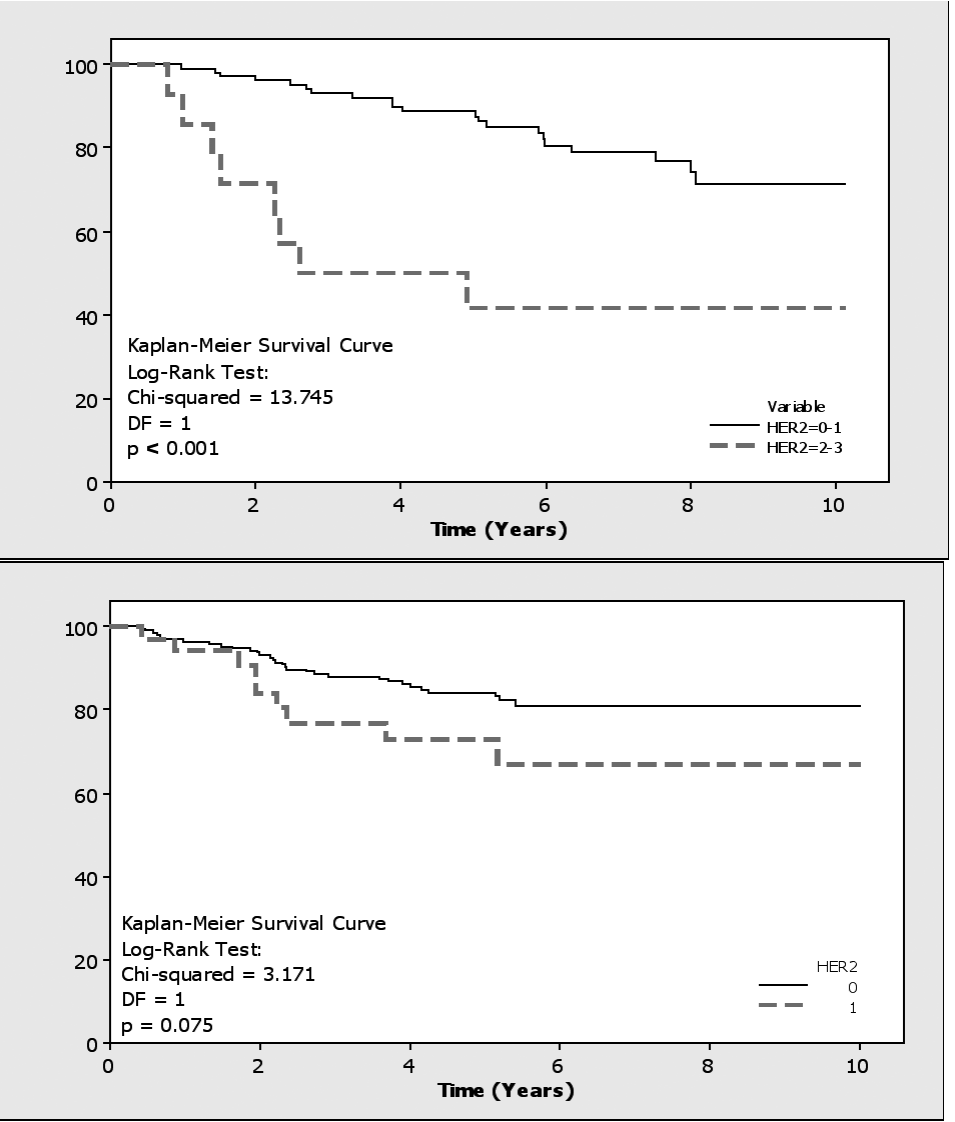
**Kaplan-Meier survival analysis correlating HER2 status to overall survival in both cohorts (p < 0.001 and = 0.075 respectively)**.

HER2 amplification, found in 11.9% (14/117) and 13.9% (32/230) of patients respectively (Figure 1D), was significantly associated with Ki67 expression (FET: p = 0.006 and 0.021 respectively), higher histological grade (FET: p = 0.073 and < 0.001 respectively), and inversely with ER status (FET: p = 0.006 and 0.001 respectively). HER2 positive patients had increased recurrence (FET: p = 0.027 and 0.001, RR = 1.72 and 1.96, OR = 3.75 and 7.2, respectively); and lower overall survival (Kaplan-Meier Log Rank Test: p < 0.001 and 0.075, RR = 2.81 and 1.99, OR = 5.19 and 2.3, respectively) (Figure 3).

**Figure 4:**
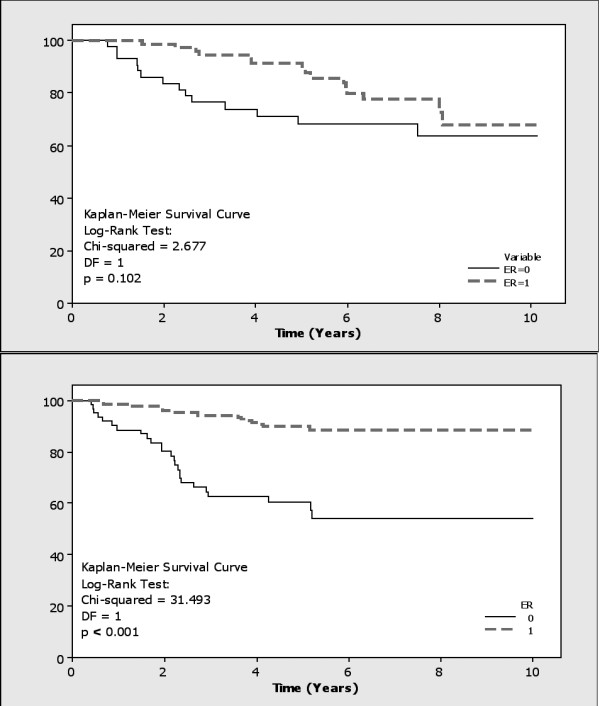
**Kaplan-Meier Survival Curve correlating ER status to overall survival in both cohorts (p = 0.102 and < 0.001 respectively)**.

ER positive tumours, identified in 62.6% (72/115) and 73% (173/237) of patients respectively, were associated with HER2 negative (FET: p = 0.006 and 0.001 respectively), low Ki67 expression (FET: p = 0.002 and < 0.001 respectively), lower grade tumours (FET: p = 0.029 and < 0.001 respectively), and lower disease recurrence (FET: p = 0.033 and < 0.001, RR = 1.41 and 2.75, OR = 2.33 and 4.36, respectively). In the second cohort of patients, ER positive patients also lived longer (Kaplan-Meier Log Rank Test: p < 0.001, RR = 1.44, OR = 5.81) (Figure 4).

## Discussion

Malignant transformation of cells is characterized by insensitivity to growth-inhibitory signals, unlimited replicative potential, angiogenesis, tissue invasion and metastasis [38]. Despite the physiological stresses in cancer cells, and the associated hypoxia in both primary and metastatic cancer [39], malignant cells continue to proliferate and invade. These hallmark characteristics of cancer raise the question of whether dysfunction of AMPK could have a role in malignancy. The aim of this study was to evaluate phosphorylation of AMPK and ACC as markers of AMPK signalling in human breast cancer in relation to clinical and pathological data.

The two cohorts of patients were representative of previously reported breast cancer populations as exemplified by the associations between HER2 amplification, Ki67 expression, ER negative cancers, higher histological grade, Ki67 labelling index [40] and higher disease recurrence and lower overall survival. The significant differences in menopausal status, age, tumour size and node status between the two cohorts (Table 1) perhaps emphasizes the consistent trends and significant associations of AMPK signalling with node status and tumour grade respectively (Table 2). Further possible caveats include the use of IHC rather than western blotting to assess the signalling pathways, although the distribution of signalling in normal and cancerous tissues on IHC allowed localisation of the proteins.

Despite the high energy consuming processes of uncontrolled cellular proliferation in breast cancer, the histological evidence suggests that AMPK does not seem to respond in primary breast cancer. In contrast, normal breast epithelium demonstrated appropriate AMPK staining, but as expected for a quiescent epithelium no activation of ACC. Reduced signalling of AMPK phosphorylation in the majority of the breast cancer specimens (89.4% in the first and 91.9% in the second cohort) compared to strong expression in normal breast epithelial cells, and the inverse relationship between staining with histological grade and node status supports the current view that activating this energy-sensing enzyme may be a potential therapeutic avenue in breast cancer [8]. Lower tumour growth fraction, assessed by lower Ki67 proliferation index in patients with activated ACC, a downstream target of AMPK, is consistent with *in vitro *evidence that AMPK activation leads to reduced proliferation [41] and growth inhibition in epithelial cells [34], also associated with decreased mTOR signalling [42,43]. AMPK activation is known to lead to inhibition of the mTOR pathway [44,45] through phosphorylation of TSC2 [32] and Raptor [33]. Activation of AMPK also directly limits translational initiation and protein synthesis through inhibition of translation elongation factor 2 (EF2) [46]. In this study we have focused on histological evidence for AMPK phosphorylation and its direct downstream target, ACC, as markers of AMPK activation, and have correlated them with the functionally important biological markers of breast cancer: ER, Ki67 and HER2.

## Conclusion

In conclusion, the role of AMPK in the mechanism of action of metformin [47-49], evidence in support of a putative relationship between type 2 diabetes and neoplasia with particular reference to breast cancer [50], and the reduced risk of cancer in patients with type 2 diabetes taking metformin compared with those on other medications such as sulphonylureas [35,51], are all indicative of the need to explore this relationship and its potential to discover a novel targeted therapy in breast cancer. AMPK activation is a possible therapeutic target for cancers, since AMPK inhibits mTOR signalling, which in turn inhibit tumour growth *in vitro *and metastasis in experimental animal models [20]. The data presented here, the first immunohistochemical evidence of AMPK dysfunction in primary breast cancer supports further laboratory and clinical research targeting AMPK by metformin or its analogues in breast cancer.

## Abbreviations

ACC: Acetyl Coenzyme-A Carboxylase; AMPK: Adeonsine Monophosphate Protein Kinase; ATP: Adensine Triphosphate; DAB: Diaminobenzidine; EDTA: Ethylene Diamine Tetra acetic Acid disodium salt dihydrate; FAS: Fatty Acid Synthase; FET: Fisher's Exact Test; FISH: Fluorescence In Situ Hybridization; IHC: Immunohistochemistry; LCFA: Long Chain Fatty Acids; mTOR: mammalian Target Of Rapamycin; NCRI: National Cancer Research Institute; OR: Odds Ratio; pACC: phospho Acetyl Coenzyme-A Carboxylase; pAMPK: phospho Adenosine Monophosphate Protein Kinase; RR: Relative Risk; TBS: Tris-Buffered Saline.

## Competing interests

The authors declare that they have no competing interests.

## Authors' contributions

SMH carried out the TMA staining, immuno-reactivity scoring, initial analysis of the data, and drafted the manuscript. KER and LJ constructed the TMAs. KER wrote the 'method of TMA preparation' part of the materials and methods, and involved in drafting the manuscript. GT and SB guided and helped SMH in TMA staining. LB analysed the data and wrote the statistics section of the manuscript. SF participated rationally in the project design, and with LJ and CAP verified TMA staining and scoring. DGH critically revised the manuscript. AMT substantially contributed to the design of the project, drafting of the manuscript and critically revised it, and gave final approval of the version to be published. All authors read and approved the final manuscript.

## Pre-publication history

The pre-publication history for this paper can be accessed here:

http://www.biomedcentral.com/1471-2407/9/307/prepub

## Supplementary Material

Additional file 1 **Supplementary Table**. This table shows the inter-relationship between the biomarkers of interest and all clinico-pathological data. Click here for file
