# Supplementary material for: Histological evaluation of AMPK signalling in primary breast cancer
Source: BMC Cancer. 2009 Sep 1;9:307. doi: 10.1186/1471-2407-9-307 (PMC2744705; doi:10.1186/1471-2407-9-307)
Supplement: Additional file 1 — Supplementary Table. This table shows the inter-relationship between the biomarkers of interest and all clinico-pathological data. [file 1471-2407-9-307-S1.doc]

**Table 3: Inter-relationship between the biomarkers of interest and all clinico-pathological data**

| **Biomarker** | **Cohort** |  | **HER2** | | | **Ki67** | | | **AMPK** | | | **ACC** | | | **ERα** | | | **Tumour size (cm)** | | | **Axillary node status** | | | **Histological grade** | | | |
| --- | --- | --- | --- | --- | --- | --- | --- | --- | --- | --- | --- | --- | --- | --- | --- | --- | --- | --- | --- | --- | --- | --- | --- | --- | --- | --- | --- |
|  |  |  | **Positive** | **Negative** | **P** | **Positive** | **Negative** | **P** | **Positive** | **Negative** | **P** | **Positive** | **Negative** | **P** | **Positive** | **Negative** | **P** | **≤ 2** | **>2** | **P** | **Positive** | **Negative** | **P** | **1** | **2** | **3** | **P** |
| **Ki67** | **1** | Positive | 10 | 33 | 0.006+ |  |  |  |  |  |  |  |  |  |  |  |  |  |  |  |  |  |  |  |  |  |  |
|  |  | Negative | 4 | 70 |  |  |  |  |  |  |  |  |  |  |  |  |  |  |  |  |  |  |  |  |  |  |  |
|  | **2** | Positive | 15 | 48 | 0.021+ |  |  |  |  |  |  |  |  |  |  |  |  |  |  |  |  |  |  |  |  |  |  |
|  |  | Negative | 17 | 135 |  |  |  |  |  |  |  |  |  |  |  |  |  |  |  |  |  |  |  |  |  |  |  |
| **AMPK** | **1** | Positive | 0 | 12 | NS | 4 | 8 | NS |  |  |  |  |  |  |  |  |  |  |  |  |  |  |  |  |  |  |  |
|  |  | Negative | 14 | 87 |  | 36 | 65 |  |  |  |  |  |  |  |  |  |  |  |  |  |  |  |  |  |  |  |  |
|  | **2** | Positive | 2 | 17 | NS | 5 | 13 | NS |  |  |  |  |  |  |  |  |  |  |  |  |  |  |  |  |  |  |  |
|  |  | Negative | 30 | 176 |  | 59 | 141 |  |  |  |  |  |  |  |  |  |  |  |  |  |  |  |  |  |  |  |  |
| **ACC** | **1** | Positive | 10 | 34 | 0.016+ | 18 | 26 | NS | 7 | 20 | 0.007+ |  |  |  |  |  |  |  |  |  |  |  |  |  |  |  |  |
|  |  | Negative | 4 | 66 |  | 23 | 47 |  | 5 | 81 |  |  |  |  |  |  |  |  |  |  |  |  |  |  |  |  |  |
|  | **2** | Positive | 14 | 40 | 0.006+ | 14 | 39 | NS | 7 | 29 | 0.014+ |  |  |  |  |  |  |  |  |  |  |  |  |  |  |  |  |
|  |  | Negative | 18 | 153 |  | 50 | 115 |  | 12 | 186 |  |  |  |  |  |  |  |  |  |  |  |  |  |  |  |  |  |
| **ERα** | **1** | Positive | 4 | 68 | 0.006- | 19 | 53 | 0.002- | 9 | 61 | NS | 30 | 41 | NS |  |  |  |  |  |  |  |  |  |  |  |  |  |
|  |  | Negative | 10 | 33 |  | 24 | 19 |  | 3 | 38 |  | 14 | 27 |  |  |  |  |  |  |  |  |  |  |  |  |  |  |
|  | **2** | Positive | 17 | 156 | 0.001- | 36 | 130 | <0.001- | 12 | 162 | NS | 37 | 137 | NS |  |  |  |  |  |  |  |  |  |  |  |  |  |
|  |  | Negative | 15 | 36 |  | 29 | 22 |  | 7 | 46 |  | 17 | 36 |  |  |  |  |  |  |  |  |  |  |  |  |  |  |
| **Tumour size (cm)** | **1** | ≤ 2 | 3 | 68 | NS | 11 | 60 | NS | 10 | 58 | 0.073- | 7 | 61 | NS | 48 | 21 | 0.045- |  |  |  |  |  |  |  |  |  |  |
|  |  | >2 | 4 | 42 |  | 11 | 35 |  | 2 | 43 |  | 5 | 41 |  | 24 | 22 |  |  |  |  |  |  |  |  |  |  |  |
|  | **2** | ≤ 2 | 21 | 140 | NS | 46 | 110 | NS | 17 | 146 | NS | 45 | 119 | 0.041- | 132 | 37 | NS |  |  |  |  |  |  |  |  |  |  |
|  |  | >2 | 11 | 53 |  | 19 | 44 |  | 2 | 63 |  | 11 | 58 |  | 49 | 18 |  |  |  |  |  |  |  |  |  |  |  |
| **Axillary node status** | **1** | Positive | 8 | 40 | NS | 22 | 26 | NS | 4 | 62 | 0.061- | 22 | 25 | NS | 25 | 22 | NS | 30 | 18 | NS |  |  |  |  |  |  |  |
|  |  | Negative | 6 | 63 |  | 21 | 48 |  | 8 | 39 |  | 22 | 45 |  | 47 | 21 |  | 41 | 28 |  |  |  |  |  |  |  |  |
|  | **2** | Positive | 19 | 86 | NS | 38 | 62 | 0.025+ | 4 | 100 | 0.039- | 24 | 82 | NS | 80 | 33 | 0.043- | 74 | 39 | 0.060- |  |  |  |  |  |  |  |
|  |  | Negative | 12 | 104 |  | 27 | 88 |  | 15 | 126 |  | 29 | 89 |  | 99 | 21 |  | 93 | 28 |  |  |  |  |  |  |  |  |
| **Histological Grade** | **1** | 1 | 0 | 12 | 0.073+ | 2 | 10 | <0.001+ | 5 | 7 | 0.010- | 3 | 9 | NS | 8 | 2 | 0.029- | 9 | 3 | 0.073+ | 5 | 7 | 0.035+ |  |  |  |  |
|  |  | 2 | 4 | 40 |  | 9 | 34 |  | 2 | 39 |  | 17 | 24 |  | 32 | 11 |  | 29 | 14 |  | 10 | 33 |  |  |  |  |  |
|  |  | 3 | 10 | 49 |  | 32 | 27 |  | 4 | 27 |  | 24 | 34 |  | 32 | 30 |  | 33 | 29 |  | 33 | 29 |  |  |  |  |  |
|  | **2** | 1 | 1 | 29 | <0.001+ | 4 | 26 | <0.001+ | 6 | 27 | 0.021- | 7 | 26 | NS | 31 | 1 | <0.001- | 28 | 4 | 0.003+ | 9 | 21 | 0.007+ |  |  |  |  |
|  |  | 2 | 2 | 86 |  | 13 | 71 |  | 7 | 81 |  | 22 | 66 |  | 88 | 4 |  | 71 | 21 |  | 39 | 52 |  |  |  |  |  |
|  |  | 3 | 28 | 74 |  | 47 | 52 |  | 5 | 99 |  | 24 | 80 |  | 60 | 47 |  | 67 | 41 |  | 62 | 45 |  |  |  |  |  |
| **KM overall survival** | **1** | Alive | 2 | 86 | <0.001- | 13 | 75 | 0.023- | 11 | 73 | NS | 7 | 78 | 0.060+ | 57 | 29 | NS | 56 | 32 | NS | 44 | 44 | <0.001- | 10 | 34 | 44 | NS |
|  |  | Dead | 5 | 24 |  | 9 | 20 |  | 1 | 28 |  | 5 | 24 |  | 15 | 14 |  | 15 | 14 |  | 25 | 4 |  | 2 | 9 | 18 |  |
|  | **2** | Alive | 22 | 162 | 0.075- | 44 | 135 | 0.015- | 15 | 172 | NS | 44 | 143 | NS | 163 | 33 | <0.001+ | 146 | 49 | 0.009- | 81 | 112 | <0.001- | 32 | 85 | 76 | <0.001- |
|  |  | Dead | 10 | 32 |  | 21 | 19 |  | 4 | 38 |  | 11 | 31 |  | 19 | 23 |  | 24 | 19 |  | 32 | 10 |  | 0 | 7 | 32 |  |

**Notes: The table includes Non-parametric Kaplan-Meier overall survival using the logrank test (all analyses have one degree of freedom).**

**(+) Denotes a positive association, and (-) a negative association. NS signifies that results are not statistically significant.**

**For each parameter the data from the first and second cohort are shown separately; first rows are the first cohort, and the second rows are the second cohort of patients.**
